# β‐Guanidinopropionic acid extends the lifespan of *Drosophila melanogaster* via an AMP‐activated protein kinase‐dependent increase in autophagy

**DOI:** 10.1111/acel.12371

**Published:** 2015-06-29

**Authors:** Si Yang, Li‐Hong Long, Di Li, Jian‐Kang Zhang, Shan Jin, Fang Wang, Jian‐Guo Chen

**Affiliations:** ^1^ Department of PharmacologySchool of Basic MedicineTongji Medical CollegeHuazhong University of Science and TechnologyWuhan430030China; ^2^ The Key Laboratory of Neurological Diseases (HUST)Ministry of Education of ChinaWuhan430030China; ^3^ Hubei Key Laboratory of Drug Target Researches and Pharmacodynamic Evaluation (HUST)Wuhan430030China; ^4^ The Laboratory of Neuropsychiatric DiseasesThe Institute of Brain ResearchHuazhong University of Science and TechnologyWuhan430030China; ^5^ College of Life ScienceHubei UniversityWuhan430062China

**Keywords:** AMP‐activated protein kinase, autophagy, autophagy‐related protein 1, β‐guanidinopropionic acid, *Drosophila*, lifespan

## Abstract

Previous studies have demonstrated that AMP‐activated protein kinase (AMPK) controls autophagy through the mammalian target of rapamycin (mTOR) and Unc‐51 like kinase 1 (ULK1/Atg1) signaling, which augments the quality of cellular housekeeping, and that β‐guanidinopropionic acid (β‐GPA), a creatine analog, leads to a chronic activation of AMPK. However, the relationship between β‐GPA and aging remains elusive. In this study, we hypothesized that feeding β‐GPA to adult *Drosophila* produces the lifespan extension via activation of AMPK‐dependent autophagy. It was found that dietary administration of β‐GPA at a concentration higher than 900 mm induced a significant extension of the lifespan of *Drosophila melanogaster* in repeated experiments. Furthermore, we found that Atg8 protein, the homolog of microtubule‐associated protein 1A/1B‐light chain 3 (LC3) and a biomarker of autophagy in *Drosophila*, was significantly upregulated by β‐GPA treatment, indicating that autophagic activity plays a role in the effect of β‐GPA. On the other hand, when the expression of Atg5 protein, an essential protein for autophagy, was reduced by RNA interference (RNAi), the effect of β‐GPA on lifespan extension was abolished. Moreover, we found that AMPK was also involved in this process. β‐GPA treatment significantly elevated the expression of phospho‐T172‐AMPK levels, while inhibition of AMPK by either AMPK‐RNAi or compound C significantly attenuated the expression of autophagy‐related proteins and lifespan extension in *Drosophila*. Taken together, our results suggest that β‐GPA can induce an extension of the lifespan of *Drosophila* via AMPK‐Atg1‐autophagy signaling pathway.

**Abbreviations**
β‐GPA: β‐guanidinopropionic acid
AMPK: AMP‐activated protein kinase
Atg1: autophagy‐related gene 1
Atg5: autophagy‐related gene 5
Atg8: autophagy‐related gene 8
mTOR: mammalian target of rapamycin
UNC: uncoordinated
ULK1: Unc‐51‐like kinase 1

## Introduction

Aging is accompanied by accumulation of cellular damage, changes in the repair and detoxification processes, and a shifting homeostatic balance in conflicting lethal and vital signaling programs (Rubinstein & Kimchi, 2012). β‐Guanidinopropionic acid (β‐GPA) is a creatine analog. A wide range of pharmacological effects of β‐GPA have been reported, including increase in mitochondrial enzyme activities and biogenesis in muscle (Reznick & Shulman, 2006; Reznick *et al*., 2007), and decrease in plasma glucose concentration and antihyperglycemic effect (Mukhina *et al*., 2008). Rush *et al*. also reported that β‐GPA decreased the content of ATP in the muscle, which then resulted in the reduction in ATP/AMP ratio and led to a chronic activation of AMP‐activated protein kinase (AMPK) (Rush *et al*., 1998).

AMP‐activated protein kinase is an evolutionarily conserved serine/threonine kinase that plays a key role in regulating the homeostasis of cellular energy in the whole body (Li & McCullough, 2010). It can be phosphorylated and activated in response to an increase in the intracellular AMP‐to‐ATP ratio during glucose deprivation, hypoxia, or ischemia and maintains cellular energy by suppressing ATP consumption as well as stimulating ATP generation (Hardie, 2003). *Drosophila melanogaster* SNF1A/dAMPKa (CG3051), a catalytic subunit of dAMPK, is a single ortholog for its mammalian and yeast counterparts (Lee *et al*., 2007). Several studies in lower organisms have revealed that increased activity of AMPK can extend the lifespan. Considering that β‐GPA can induce a chronic activation of AMPK, we asked whether β‐GPA can extend the lifespan of *Drosophila melanogaster*.

Recent studies have revealed that autophagy has been shown to play an important role for lifespan extension by treatment with spermidine (Eisenberg *et al*., 2009), rapamycin (Alvers *et al*., 2009b), or resveratrol (Morselli *et al*., 2010), as well as by depletion of the p53 ortholog from Caenorhabditis elegans, the inhibition of IGF signaling, and the overexpression of sirtuin (Tavernarakis *et al*., 2008). The reduction in autophagy activity has been observed in a number of aging models, and its upregulation via pharmacological or genetic methods can alleviate age‐related disorders and extend lifespan. Moreover, autophagy induction can enhance clearance of toxic intracellular waste associated with neurodegenerative diseases and has been demonstrated to improve lifespan in yeast, worms, flies, and rodents (Lunell *et al*., 1991; Eisenberg *et al*., 2009; Carroll *et al*., 2013; Babcock *et al*., 2015). As a conserved process participating bioenergetic management by degrading and recycling cellular constituents, autophagy has been recently proposed as a downstream target of AMPK (Wong *et al*., 2009). Experiments in mammals have demonstrated that AMPK controls autophagy through mammalian target of rapamycin (mTOR) and Atg1/ULK1 signalings, which augments the quality of cellular housekeeping (Alers *et al*., 2012). Previous studies indicate that the activation of AMPK is able to induce autophagy and subsequently confer to the protective effects against ischemic injury in the heart (Matsui *et al*., 2007), kidney (Wang *et al*., 2013), liver (Zaouali *et al*., 2013), and muscular tissues (Pauly *et al*., 2012). In the present study, we investigated the effects of β‐GPA in dietary supplements on lifespan of *Drosophila* and the underlying mechanisms. The results can provide new evidence or clues for developing new drug for anti‐aging or food to extend lifespan.

## Results

### β*‐GPA* extends lifespan and increases stress resistance in *Drosophila*

**Figure 1:**
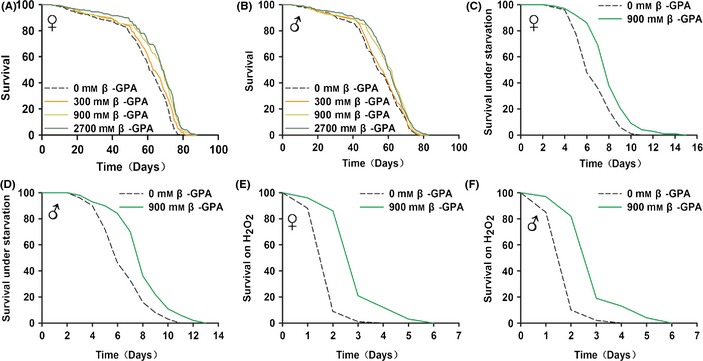
β‐guanidinopropionic acid (GPA) treatment extends lifespan and healthspan in *Drosophila*. (A, B) β‐GPA treatment extends the lifespan of females and males. Compared to flies with control food (0 mm β‐GPA), flies on 900 and 2700 mm β‐GPA food had increased median lifespan (*n* = 200, *P* < 0.001, log‐rank test). (C, D) Flies were pretreated for 30 days with 900 mm β‐GPA. β‐GPA enhances starvation resistance of wild‐type (WT) females and males (*n* = 100, *P* < 0.001, log‐rank test). (E, F) Flies were pretreated for 30 days with 900 mm β‐GPA. β‐GPA‐pretreated flies had improved survival on 3 M H_2_O_2_ both in WT females and males (*n* = 100, *P* < 0.001, log‐rank test).

To determine whether β‐GPA can extend lifespan in *Drosophila*, the flies were fed in a normal chow diet containing different concentrations of β‐GPA. To avoid developmental effects, fruit flies were raised on normal food for 7 days after brooding, and then fed a diet supplemented with β‐GPA at three different concentrations, when comparable to the effective dose administered to mice (Bergeron *et al*., 2001). We found no significant effect of β‐GPA on the amount of food consumed by using a blue‐dye feeding assay (Figure S1). As shown in Fig. 1, the median lifespan in both male and female *Drosophila* was significantly increased by β‐GPA at either 900 mm (females: 60 days (control) vs. 68 days (β‐GPA); males: 55 days (control) vs. 59 days (β‐GPA), *n* = 200, *P* < 0.001, log‐rank test) or 2700 mm (females: 60 days (control) vs. 68 days (β‐GPA); males: 55 days (control) vs. 60 days (β‐GPA), *n* = 200, *P* < 0.001, log‐rank test), indicating that β‐GPA produces a significant effect on lifespan extension (Fig. 1A,B). However, β‐GPA at lower concentration (300 mm) had little effect. There were no significant difference between 900 mm and 2700 mm β‐GPA.

Interventions that increase the lifespan of *Drosophila* are often associated with resistance to various stresses (Clancy *et al*., 2001; Broughton *et al*., 2005). Thus, we examined the survival of β‐GPA‐treated flies under starvation or oxidative stress induced by H_2_O_2_ (Zhang *et al*., 2014). The *Drosophila* were pretreated with 900 mm β‐GPA for 30 days and then transferred either to agar‐only food for starvation assays or to food containing 3 m H_2_O_2_. The results showed that pretreatment with β‐GPA significantly increased the median lifespan under starvation in both male and female *Drosophila* (females: 5 days (control) vs. 7 days (β‐GPA); males: 5 days (control) vs. 7 days (β‐GPA), *n* = 100, *P* < 0.001, log‐rank test, Fig. 1C,D). β‐GPA also increased the median lifespan under H_2_O_2_‐treated *Drosophila* (females: 1 days (control) vs. 2 days (β‐GPA); males: 1 days (control) vs. 2 days (β‐GPA), *n* = 100, *P* < 0.001, log‐rank test, Fig. 1E,F), indicating that β‐GPA increases the resistance of *Drosophila* to various stresses.

### β*‐*GPA produces longevity via promoting autophagy of *Drosophila*

**Figure 2:**
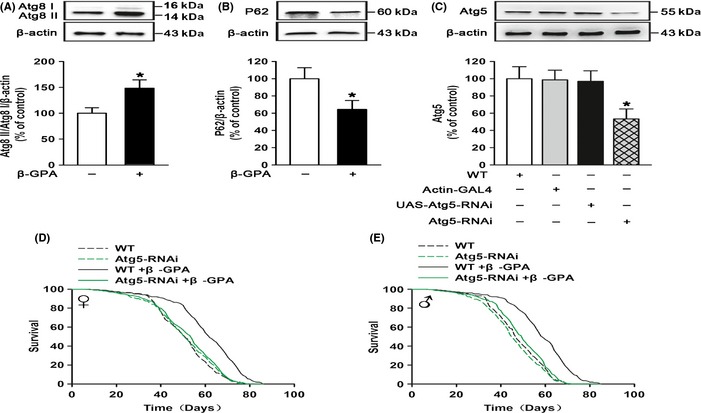
β‐guanidinopropionic acid (GPA) promotes autophagy that is essential for longevity in *Drosophila*. Western blot analysis of Atg8 in wild‐type (WT) flies. β‐GPA was found to efficiently increase the levels of Atg8 in flies (*n* = 6, one‐way ANOVA, *P < 0.05 vs. control). (B) The levels of P62 were downregulated upon β‐GPA administration (*n* = 6, *P* < 0.05 vs. control, Student's *t*‐test). (C) Western blot analysis of Atg5 in actin‐GAL4>UAS‐Atg5‐RNAi flies (Atg5‐RNAi). Atg5 was reduced in Atg5‐RNAi flies (*n* = 6, one‐way ANOVA, *P < 0.05 vs. control). (D, E) Downregulation of autophagy abolished the β‐GPA‐mediated lifespan extension (*n* = 200, *P* < 0.0001 vs. control, log‐rank test). For all Western blots, relative band intensity was estimated using IMAGEJ. Data are shown as mean ± SEM.

It has been demonstrated that autophagy might be one of the major pathways responsible for lifespan extension under certain circumstances in various organisms (Rubinsztein *et al*., 2011). Then, we asked whether β‐GPA could increase autophagy in the *Drosophila*. The levels of Atg8 (a biomarker of autophagy) and P62 were measured by Western blot analysis using anti‐Atg8 and anti‐P62 antibodies. Figure 2A shows that the levels of Atg8 II/Atg8 I were upregulated significantly after 900 mm β‐GPA treatment for 30 days (control: 100 ± 10.64, β‐GPA: 148.43 ± 16.01, *n* = 6, *P* < 0.05 vs. control, Student's *t*‐test). We also observed that the levels of P62 were downregulated upon β‐GPA administration (control: 100 ± 12.58, β‐GPA: 64.37 ± 10.39, *n* = 6, *P* < 0.05 vs. control, Student's *t*‐test, Fig. 2B). Taken together, these data confirm that β‐GPA increases autophagy *in vivo*.

Furthermore, we examined whether the extension of lifespan by β‐GPA was mediated by the upregulation of autophagy. It is well known that Atg5 is required for formation of autophagosomes and is specific and essential for autophagy (Reggiori & Klionsky, 2005). We then applied RNA interference to knock down the expression of Atg5. As shown in Fig. 2C, the expression of Atg5 was downregulated by ubiquitous overexpression of UAS‐Atg5‐RNAi using actin‐GAL4 (actin‐GAL4>UAS‐Atg5‐RNAi, Atg5‐RNAi) (one‐way ANOVA, *F*(3, 20) = 3.394, *P* < 0.05). Subsequent *post hoc* analysis indicated that the protein of Atg5 was significantly downregulated when compared to control (control: 100 ± 13.89, Atg5‐RNAi: 53.37 ± 11.57, *n* = 6, *P* < 0.05 vs. control). Strikingly, the lifespan extension (median lifespan) induced by β‐GPA was simultaneously prevented by actin‐GAL4>UAS‐Atg5‐RNAi (females: 62 days (β‐GPA) vs. 51 days (Atg5‐RNAi + β‐GPA); males: 59 days (β‐GPA) vs. 51 days (Atg5‐RNAi + β‐GPA), *n* = 200, *P* < 0.001, log‐rank test, Fig. 2D,E), suggesting that the activation of autophagy is required for the lifespan extension by β‐GPA treatment.

### β*‐*GPA increases the activity of AMPK in *Drosophila*

**Figure 3:**
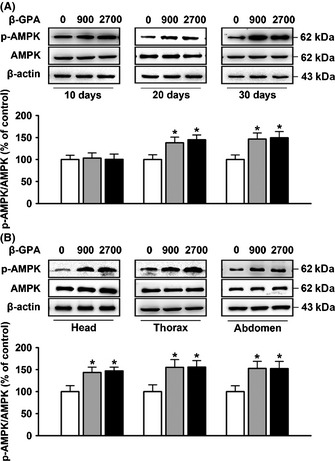
β‐guanidinopropionic acid (GPA) treatment upregulates AMP‐activated protein kinase (AMPK) activity in *Drosophila*. (A) Western blot analysis of phospho‐AMPK on whole‐fly protein extracts. Flies were sampled after 10, 20, or 30 days of β‐GPA treatment at concentrations of 0 (control), 900, or 2700 mm. A time‐dependent upregulation in phospho‐T172‐AMPK levels was observed (*n* = 6, one‐way ANOVA, *P < 0.05 vs. control). (B) Western blot analysis of phospho‐AMPK in heads, thoraces, and abdomens, respectively. Flies were maintained with or without 900 mm β‐GPA for 30 days prior to preparation of protein extracts from heads, thoraces, and abdominal segments. β‐GPA was found to efficiently increase the levels of phospho‐T172‐AMPK in heads, thoraces, and abdomens (*n* = 6, one‐way ANOVA, *P < 0.05 vs. control). For all Western blots, relative band intensity was estimated using imagej. All data are shown as mean ± SEM.

It was reported that β‐GPA can induce a chronic activation of AMPK by increasing the AMP/ATP ratio in mice (Bergeron *et al*., 2001). To investigate whether β‐GPA can also increase the AMPK activity in *Drosophila*, we measured the AMPK activity in β‐GPA‐fed *Drosophila* at concentrations of 300, 900, and 2700 mm and found no effect of β‐GPA (treated for 30 days) on phosphorylation of AMPK at doses of 300 mm (Figure S2). As shown in Fig. 3A, a significant elevated expression of phospho‐T172‐AMPK levels in *Drosophila* was observed after the treatment with β‐GPA for 20 days (one‐way ANOVA, *F*(2, 15) = 4.393, *P* < 0.05) and 30 days (one‐way ANOVA, *F*(2, 15) = 4.712, *P* < 0.05) at doses of 900 and 2700 mm (control: 100 ± 10.82, 900 mm: 137.93 ± 12.83, 2700 mm: 144.97 ± 10.85 after 20 days; control: 100 ± 10.38, 900 mm: 146.58 ± 13.74, 2700 mm: 149.54 ± 13.96 after 30 days, *n* = 6, *P* < 0.05 vs. control). However, there were no effects by treatment with β‐GPA for 10 days (control: 100 ± 9.73; 900 mm: 103.24 ± 11.69; 2700 mm: 100.51 ± 11.81; *n* = 6; one‐way ANOVA, *F*(2, 15) = 0.002, *P* = 0.998). We also found that 900 mm β‐GPA can increase the AMPK activity after 50 days, when the lifespan curves start to separate (Figure S3). To determine the universality of β‐GPA throughout the *Drosophila*, we measured AMPK phosphorylation in different regions of the body. As shown in Fig. 3B, phospho‐T172‐AMPK levels were increased to similar levels in heads [one‐way ANOVA, *F*(2, 15) = 5.076, *P* < 0.05], thoraces [one‐way ANOVA, *F*(2, 15) = 4.403, *P* < 0.05], and abdomens [one‐way ANOVA, *F*(2, 15) = 4.025, *P* < 0.05] after the treatment with 900 and 2700 mm β‐GPA for 30 days (control: 100 ± 13.54, 900 mm: 143.36 ± 12.2, 2700 mm: 147.13 ± 8.54 in heads; control: 100 ± 15.49, 900 mm: 155.14 ± 17.53, 2700 mm: 155.78 ± 14.59 in thoraces; control: 100 ± 13.35, 900 mm: 152.91 ± 15.8, 2700 mm: 152.4 ± 16.14 in abdomens, *n* = 6; *P* < 0.05 vs. control), suggesting that the activity of AMPK is ubiquitously upregulated in *Drosophila* upon β‐GPA treatment.

### Activation of AMPK mediates β*‐*GPA‐induced autophagy and longevity in *Drosophila*

**Figure 4:**
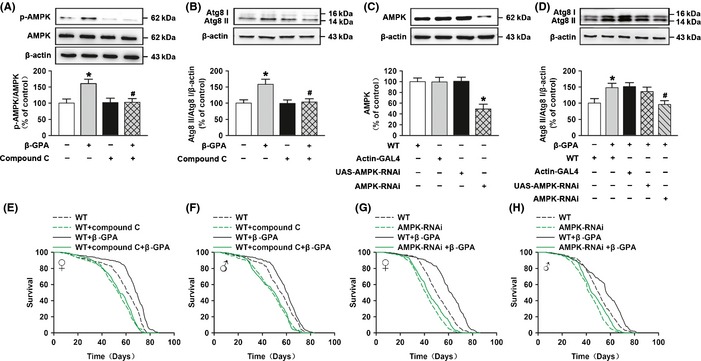
AMP‐activated protein kinase (AMPK) is important to autophagy and longevity induced by β‐guanidinopropionic acid (GPA). (A, B) Western blot analysis of phospho‐AMPK and Atg8 expression in non‐β‐GPA‐treated control, β‐GPA‐treated flies with or without compound C (pretreated for 14 days, 200 μm), and non‐β‐GPA‐treated flies with compound C. The graph indicates that β‐GPA increases phospho‐T172‐AMPK and Atg8 expression, which is prevented by the AMPK inhibitor compound C (*n* = 6, two‐way ANOVA, *P < 0.05 vs. control, ^#^ *P* < 0.05 vs. β‐GPA). (C) The levels of AMPK were decreased in actin‐GAL4>UAS‐AMPK‐RNAi (AMPK‐RNAi) flies detected by Western blotting (*n* = 6, one‐way ANOVA, *P < 0.05 vs. control). (D) Western blot analysis showed that AMPK‐RNAi significantly prevented β‐GPA‐induced expression of Atg8 protein (*n* = 6, two‐way ANOVA, *P < 0.05 vs. control, ^#^ *P* < 0.05 vs. β‐GPA). For all Western blots, relative band intensity was estimated using imagej. Data are shown as mean ± SEM. (E, F) β‐GPA does not extend the lifespan of compound C‐treated flies (*n* = 200, *P* < 0.0001 vs. control, log‐rank test). (G, H) β‐GPA does not extend the lifespan of AMPK‐RNAi flies (*n* = 200, *P* < 0.0001 vs. control, log‐rank test).

Recent studies have addressed a new mechanism for the control of mammalian autophagy by AMPK that AMPK regulates autophagy through direct phosphorylation of ULK1 (Alers *et al*., 2012). To determine the role of AMPK activation in the effect of β‐GPA on lifespan in *Drosophila*, compound C (an AMPK inhibitor) and RNA interference were employed. Firstly, the *Drosophila* were pretreated with compound C (200 μm) for 14 days and then exposed to 900 mm β‐GPA for 30 days. We found that compound C prevented the upregulation of phospho‐T172‐AMPK level induced by β‐GPA (Fig. 4A), and two‐way ANOVA indicated a significant effect of β‐GPA [*F*(1, 20) = 5.769, *P* < 0.05] and compound C [*F*(1, 20) = 4.799, p < 0.05]. Post hoc analysis showed that the level of phospho‐T172‐AMPK was increased by β‐GPA (control: 100 ± 12.76, β‐GPA: 160.82 ± 13.39, *n* = 6; *P* < 0.05 vs. control), and this effect was attenuated by compound C (β‐GPA: 160.82 ± 13.39, compound C + β‐GPA: 102.7 ± 11.66, *n* = 6; *P* < 0.05 vs. β‐GPA). In addition, we found that compound C attenuated the elevated level of Atg8 II/Atg8 I induced by β‐GPA in *Drosophila* (Fig. 4B), and two‐way ANOVA indicated a significant effect of β‐GPA [*F*(1, 20) = 6.879, *P* < 0.05] and compound C [*F*(1, 20) = 5.852, *P* < 0.05]. β‐GPA increased the level of Atg8 II/Atg8 I (control: 100 ± 10.5, β‐GPA: 158.53 ± 15.71, *n* = 6, *P* < 0.05 vs. control); however, this effect was inhibited by compound C (β‐GPA: 158.53 ± 15.71, compound C + β‐GPA: 103.52 ± 10.25, *n* = 6, *P* < 0.05 vs. β‐GPA). Meanwhile, compound C prevented the extension of median lifespan induced by β‐GPA (females: 68 days (β‐GPA) vs. 54 days (compound C + β‐GPA); males: 60 days (β‐GPA) vs. 50 days (compound C + β‐GPA), *n* = 200, *P* < 0.001, log‐rank test, Fig. 4E,F).

Furthermore, the ubiquitous double‐stranded RNA interference was used against α subunit of AMPK (AMPKα), and the manipulation resulted in a reduction in the expression of AMPK [one‐way ANOVA, *F*(3, 20) = 10.186, *P* < 0.001, Fig. 4C]. Subsequent post hoc analysis indicated that the level of AMPK was significantly downregulated when compared to control (control: 100 ± 6.87, AMPK‐RNAi: 49.08 ± 8.92, *n* = 6, *P* < 0.001 vs. control). AMPK‐RNAi also attenuated the activation of autophagy induced by β‐GPA (Fig. 4D), and two‐way ANOVA indicated a significant effect of β‐GPA [*F*(1, 25) = 6.721, *P* < 0.05] and AMPK‐RNAi [*F*(3, 25) = 3.203, *P* < 0.05]. Post hoc analysis showed that the level of Atg8 II/Atg8 I was increased by β‐GPA (control: 100 ± 14.29, β‐GPA: 147.95 ± 13.52, *n* = 6, *P* < 0.05 vs. control), and this effect was attenuated by AMPK‐RNAi (β‐GPA: 147.95 ± 13.52, AMPK‐RNAi + β‐GPA: 96.05 ± 11.8, *n* = 6, *P* < 0.05 vs. β‐GPA). AMPK‐RNAi completely prevented the extension of lifespan by β‐GPA (median lifespan for females: 63 days (β‐GPA) vs. 51 days (AMPK‐RNAi + β‐GPA), and for males: 57 days (β‐GPA) vs. 51 days (AMPK‐RNAi + β‐GPA), *n* = 200, *P* < 0.001, log‐rank test, Fig. 4G,H), suggesting that the activation of AMPK plays an important role in β‐GPA‐induced autophagy and lifespan extension in *Drosophila*.

### AMPK‐induced phosphorylation of Atg1 is essential for the lifespan extension induced by β‐GPA

**Figure 5:**
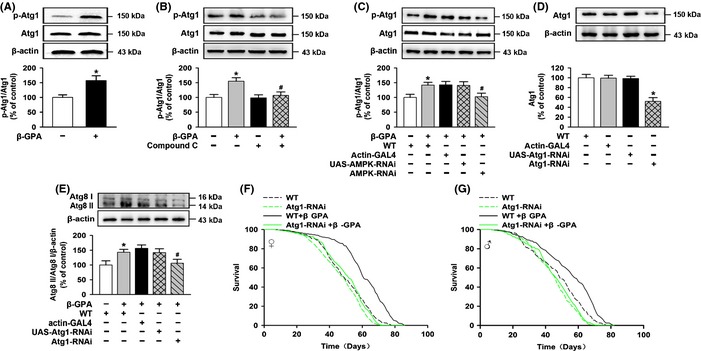
AMP‐activated protein kinase (AMPK)‐induced phosphorylation of ULK1 is essential for the lifespan extension induced by β‐guanidinopropionic acid (GPA). (A) Phospho‐Atg1 protein was detected by Western blotting of flies fed β‐GPA. Phospho‐S555‐Atg1 expression was increased by β‐GPA (*n* = 6, one‐way ANOVA, *P < 0.05 vs. control). (B) Western blot analysis of phospho‐Atg1 expression in non‐β‐GPA‐treated control, β‐GPA‐treated flies with or without compound C (pretreated for 14 days, 200 μm), and non‐β‐GPA‐treated flies with compound C. The graph indicates that β‐GPA increases phospho‐S555‐Atg1 expression, which is prevented by the AMPK inhibitor compound C (*n* = 6, two‐way ANOVA, *P < 0.05 vs. control, ^#^ *P* < 0.05 vs. β‐GPA). (C) Western blot analysis showed that AMPK‐RNAi significantly prevented β‐GPA‐induced expression of phospho‐S555‐Atg1 protein (*n* = 6, two‐way ANOVA, *P < 0.05 vs. control, ^#^ *P* < 0.05 vs. β‐GPA). (D) The levels of Atg1 were decreased in actin‐GAL4>UAS‐Atg1‐RNAi (Atg1‐RNAi) flies tested by Western blotting (*n* = 6, one‐way ANOVA, *P < 0.05 vs. control). (E) Western blot analysis showed that Atg1‐RNAi significantly prevented β‐GPA‐induced expression of Atg8 protein (*n* = 6, two‐way ANOVA, *P < 0.05 vs. control, ^#^ *P* < 0.05 vs β‐GPA). For all Western blots, relative band intensity was estimated using imagej. Data are shown as mean ± SEM. (F, G) Downregulation of Atg1 abolished β‐GPA‐mediated lifespan extension (*n* = 200, *P* < 0.0001 vs. control, log‐rank test).

It has been reported that AMPK directly phosphorylates and activates Atg1 to initiate autophagy (Egan *et al*., 2011), and the Atg1 homolog in *Drosophila* is UNC‐51‐like kinase 1 (ULK1). To test the role of Atg1 phosphorylation induced by AMPK in the effects of β‐GPA, we fed *Drosophila* with β‐GPA for 30 days. Then, we measured the levels of phosphorylation of Atg1 by Western blotting using phospho‐S555‐Atg1 antibody. Figure 5A shows that β‐GPA treatment for 30 days induced a significant increase in the phosphorylation of Atg1 (control: 100 ± 8.75, β‐GPA: 157.29 ± 16.17, *n* = 6, Student's *t*‐test, *P* < 0.05 vs. control), indicating that β‐GPA increases the phosphorylation of Atg1 in *Drosophila*.

Next, to investigate the association of activated AMPK with activation of Atg1, AMPK inhibitor compound C and RNA interference were employed. We found that when pretreated with compound C (200 μm) for 14 days in *Drosophila*, β‐GPA treatment failed to increase the phosphorylation of Atg1 in *Drosophila* (Fig. 5B). Two‐way ANOVA indicated a significant effect of β‐GPA [*F*(1, 20) = 8.148, *P* < 0.05] and compound C [*F*(1, 20) = 5.016, *P* < 0.05]. While β‐GPA increased the phosphorylation of Atg1 (control: 100 ± 9.91, β‐GPA: 154.92 ± 11.96, *n* = 6, *P* < 0.05 vs. control), compound C treatment significantly prevented the elevated phosphorylation of Atg1 induced by β‐GPA (β‐GPA: 154.92 ± 11.96, compound C + β‐GPA: 106.79 ± 11.84, *n* = 6, *P* < 0.05 vs. β‐GPA). Similar effect was observed in AMPK‐RNAi *Drosophila*, and we found that when RNA interference of AMPK was used in *Drosophila*, β‐GPA treatment failed to increase the phosphorylation of Atg1 in *Drosophila* (Fig. 5C). Two‐way ANOVA indicated a significant effect of β‐GPA [*F*(1, 25) = 6.834, *P* < 0.05] and AMPK‐RNAi [*F*(3, 25) = 3.026, *P* < 0.05]. Post hoc analysis showed that the phosphorylation of Atg1 was increased by β‐GPA (control: 100 ± 10.7, β‐GPA: 142.03 ± 9.31, *P* < 0.05 vs. control, *n* = 6), while this effect was attenuated by AMPK‐RNAi (β‐GPA: 142.03 ± 9.31, AMPK‐RNAi + β‐GPA: 102.23 ± 12.16, *n* = 6, *P* < 0.05 vs. β‐GPA).

To address whether the activation of Atg1 mediated β‐GPA‐induced autophagy, Atg1 RNAi was employed. As shown in Fig. 5D, this manipulation resulted in a reduction in Atg1 expression [one‐way ANOVA, *F*(3, 20) = 14.026, *P* < 0.001]. Subsequent post hoc analysis indicated that the expression of Atg1 protein was significantly downregulated when compared to control (control: 100 ± 6.86, Atg1‐RNAi: 51.99 ± 7.61, *n* = 6, *P* < 0.001 vs. control). Atg1‐RNAi attenuated the activation of AMPK‐dependent autophagy induced by β‐GPA in *Drosophila* (Fig. 5E), and two‐way ANOVA indicated a significant effect of β‐GPA [*F*(1, 25) = 5.982, *P* < 0.05] and Atg1‐RNAi [*F*(3, 25) = 3.002, *P* < 0.05]. Post hoc analysis showed that the level of Atg8 II/Atg8 I was increased by β‐GPA (control: 100 ± 14.31, β‐GPA: 143.3 ± 9.82, *n* = 6, *P* < 0.05 vs. control), and this effect was attenuated by Atg1‐RNAi (β‐GPA: 143.3 ± 9.82, Atg1‐RNAi + β‐GPA: 105.91 ± 13.53, *n* = 6, *P* < 0.05 vs. β‐GPA). These results suggest that Atg1 is essential for β‐GPA‐induced autophagy.

Finally, we wondered whether the autophagy‐relevant activation of Atg1 participated in the extension of lifespan induced by β‐GPA. Intriguingly, Atg1‐RNAi significantly inhibited the extension of lifespan by β‐GPA (median lifespan for females 61 days (β‐GPA) vs. 50 days (Atg1‐RNAi + β‐GPA) and for males 58 days (β‐GPA) vs. 49 days (Atg1‐RNAi + β‐GPA), *n* = 200, *P* < 0.001, log‐rank test, Fig. 5F,G), indicating that Atg1 may be responsible for lifespan extension induced by β‐GPA.

### β‐GPA decreases the levels of glycolysis

**Figure 6:**
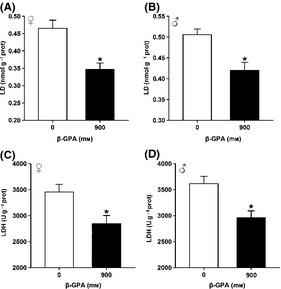
β‐guanidinopropionic acid (GPA) treatment downregulates glycolysis in *Drosophila*. (A, B) β‐GPA treatment decreased lactic acid (LD) levels. LD levels in wild‐type (WT) females and males were measured after 30 days of β‐GPA treatment. Data are shown as mean ± SEM (*n* = 6, *t*‐test, *P < 0.05 vs. control). (C, D) β‐GPA treatment decreased the lactate dehydrogenase (LDH) activity. The LDH activity in WT females and males was measured after 30 days of β‐GPA treatment. Data are shown as mean ± SEM (*n* = 6, *t*‐test, *P < 0.05 vs. control).

To determine the effect of β‐GPA on switch from glycolysis to oxidative metabolism, we tested the levels of glycolysis. The levels of glycolysis are usually determined by measuring the indicators of oxidative stress, such as lactic acid (LD) and lactate dehydrogenase (LDH). Lactic acid is a specific product in the process of glycolysis, and LDH is an important enzyme which regulates the process of glycolysis. Then, we tested the level of LD and LDH in β‐GPA‐treated *Drosophila*. The results showed that a 30‐day exposure of β‐GPA (900 mm) caused a decrease in LD content (mmol per gprot) (control: 0.47 ± 0.06, β‐GPA: 0.37 ± 0.04 in females, control: 0.51 ± 0.03, β‐GPA: 0.42 ± 0.05 in males, *n* = 6, *P* < 0.05 vs. control, *t*‐test; Fig. 6A,B) and an decrease in the activity of LDH (U per gprot) in both females and males when compared to the control (control: 3455.38 ± 356.23, β‐GPA: 2845.71 ± 385.65 in females, control: 3615.38 ± 347.11, β‐GPA: 2958.70 ± 133.56 in males, *n* = 6, *P* < 0.05 vs. control, *t*‐test; Fig. 6C,D). These results suggest that β‐GPA decreases the levels of glycolysis.

### β*‐*GPA extends lifespan beyond the maximum achieved by dietary restriction

As the activity of AMPK could be increased by dietary restriction (DR) (Silvestre *et al*., 2014), which also extends lifespan and promotes metabolic health in evolutionary distinct species (Schmeisser *et al*., 2013), we examined whether DR is involved in the lifespan effect of β‐GPA. We found no significant effect of β‐GPA on feeding behavior or on the amount of food consumed (Figure S1), and β‐GPA extends lifespan beyond the maximum achieved by DR (Figure S4). Thus, β‐GPA does not reduce food palatability and causes DR.

## Discussion

In the present study, we demonstrated that orally administered β‐GPA increased the lifespan of *Drosophila*, and this effect was mediated by the activation of AMPK and then induced autophagy in *Drosophila*. Furthermore, we found that the β‐GPA‐evoked autophagy was dependent on the activation of Atg1. Our results provide the novel evidence for the possibility to develop β‐GPA or its analogs as an anti‐aging drug or an active component of food.

Guanidinopropionic acid feeding has been shown to increase mitochondrial biogenesis by elevated levels of PGC‐1α mRNA expression, mitochondrial density, δ‐ALAS mRNA expression, and cytochrome c protein expression (Reznick *et al*., 2007). Our results demonstrate that β‐GPA decreases the level of LD and LDH, indicating the decreased glycolysis. Thus, β‐GPA stimulates the switch from glycolysis to oxidative metabolism. In addition, β‐GPA at a dose of 900 mm also enabled *Drosophila* to survive longer in periods of starvation, suggesting that β‐GPA may affect nutrient supply and available energy with cellular demands; thus, β‐GPA increases both lifespan and healthspan in *Drosophila*.

Previous studies have reported that several signaling pathways are involved in the regulation of aging process, promoting longevity in yeast, nematodes, and *Drosophila* (Salminen & Kaarniranta, 2012). For example, the reduction in insulin/IGF‐1 signaling promotes longevity in Caenorhabditis elegans and flies (Lin *et al*., 2001). Dietary restriction, a reduction in total food intake, has been shown to increase lifespan and reduce fecundity by limiting metabolic rates and the availability of metabolic energy in a wide range of organisms such as yeast, nematodes, flies, and rodents (Masoro, 2005; Mair & Dillin, 2008). Autophagic activity decreases during the course of aging, and genes that control this process are strongly associated with lifespan regulation in flies and worms (Lionaki *et al*., 2013). In *Drosophila*, overexpression of autophagy‐related genes in neurons enhances longevity, while their repression causes neuronal defects and shortening of lifespan (Simonsen *et al*., 2008). Reduced signaling through the target of rapamycin (TOR) kinase has been shown to extend lifespan in *Drosophila* (Kapahi *et al*., 2004). These pathways have many common characteristics; for example, AMPK can regulate the function of all these pathways, and many of them target the regulation of autophagy and oxidative stress, functional hallmarks of the aging process (Salminen & Kaarniranta, 2012). Autophagy is known to play a crucial role in cells that are stressed by damage or limited nutrient supply (Alvers *et al*., 2009a). We wondered whether autophagy is also involved in the longevity effect of β‐GPA. The autophagy pathway is conserved from yeasts to *Drosophila* to humans and involves more than 20 proteins, for example Atg1 through Atg27 in yeast (Baehrecke, 2003; Deretic, 2005; Mizushima *et al*., 2008). In our study, we found that chronic β‐GPA treatment can induce the level of Atg8, a biomarker of autophagy in *Drosophila*. The inhibition of autophagy by Atg5‐RNAi prevented the effects of β‐GPA on the lifespan. However, it is interesting to note that although autophagy seems to be an important contributor to longevity (Toth *et al*., 2008), we did not observe shortening of lifespan upon reduced expression of Atg5. This may be due to the fact that under our laboratory conditions, the lifespan of adult fly may be affected by some other factors. This effect was similar to that in the previous reports (Ren *et al*., 2009). Collectively, these results suggest that autophagy is required for chronic β‐GPA‐induced longevity.

Emerging evidence indicates that AMPK plays a key role in the regulation of autophagy. The finding that β‐GPA can activate AMPK in the brain is consistent with previous reports showing that β‐GPA increases the AMPK activity in peripheral tissues, such as skeletal muscle (Reznick *et al*., 2007). We found that β‐GPA upregulated the AMPK activity in *Drosophila*, and this upregulation was ubiquitous, as the levels of phosphorylated AMPK were similarly upregulated in all main segments of *Drosophila*, such as heads, thoraces, and abdomens. Notably, chronic treatment of β‐GPA 7 days after brooding resulted in a robust and repeatable extension of lifespan, which was independent of the sex of *Drosophila*.

The serine/threonine kinase ULK1 is a mammalian homolog of Atg1, a part of the ULK1/Atg1 kinase complex, which is the most upstream component of the core autophagy machinery conserved from yeast to mammals (Egan *et al*., 2011). Our study showed that the elevated levels of autophagy and lifespan extension induced by β‐GPA were abolished by Atg1‐RNAi. ULK1/Atg1‐dependent autophagy is regulated by the mammalian target of rapamycin complex 1 (mTORC1) and AMPK (Alers *et al*., 2012). Notably, the activity of ULK1/Atg1 kinase increases in an AMPK‐dependent manner following glucose deprivation (Alers *et al*., 2012). Recent studies demonstrate that AMPK directly phosphorylates ULK1/Atg1 on several sites, and this phosphorylation is required for ULK1/Atg1 activation after glucose deprivation (Alers *et al*., 2012). On the contrary, when nutrients are plentiful, the mTORC1 complex phosphorylates ULK1/Atg1, preventing the association and activation of ULK1/Atg1 by AMPK (Alers *et al*., 2012). These studies have revealed a molecular mechanism of regulation on ULK1/Atg1 by nutrient signals via the actions of AMPK and mTORC1. In the present study, we found that the activation of AMPK induced by β‐GPA inhibited mTORC1 activity (Figure S5), implicating that mTORC1 may not prevent the activation of ULK1/Atg1 by AMPK.

In our daily life, β‐GPA is used as a dietary supplement, surface active agent for cosmetics, nutrition fortifier, and so on. Previous studies show that β‐GPA and its derivatives guanidinoacetic acid (GAA) and 4‐guanidinobutyric acid (4‐GBA) decrease the concentration of plasma glucose and exert antihyperglycemic effect (Metzner *et al*., 2009). In this study, we demonstrated that dietary application of β‐GPA can extend the lifespan of *Drosophila* and this effect is mediated by AMPK‐dependent autophagy. Our results also indicate that its lifespan extension effect is not due to DR induced by β‐GPA. Considering that aging is also tightly associated with neurodegenerative disease, such as Alzheimer's disease, β‐GPA or its catalogs can also be developed into a therapeutic drug for the treatment of age‐associated diseases.

## Materials and methods

### Materials

β‐GPA, compound C, and H_2_O_2_ were purchased from Sigma‐Aldrich (St. Louis, MO, USA). The reagent kits for determining LD and LDH were purchased from Nanjing Jiancheng Institute of Biological Engineering (Nanjing, China). Primary antibodies of AMPK, p‐AMPK, ULK1 (Atg1), p‐ULK1 (p‐Atg1), Atg8, P62, S6K, and p‐S6K were purchased from Cell Signaling Technology Inc. (San Francisco, CA, USA). Atg5 was purchased from Novus Biologicals (Denver, CO, USA). Horseradish peroxidase (HRP)‐conjugated secondary antibodies were purchased from Millipore (Carrigtwohill, Co. Cork, Ireland). Other general agents were commercially available.

### *Drosophila* strains

The wild‐type stock w^1118^ (WT) was obtained from Dr. Jin Shan. Actin‐GAL4, UAS‐Atg1‐RNAi, UAS‐Atg5‐RNAi, and UAS‐AMPK‐RNAi were procured from the Tsinghua Fly Center (THFC). The *Drosophila* strain w^1118^ was used in all control crosses and as the background for the generation of transgenic lines. All stocks were maintained and all the experiments were conducted at 25 °C on a 12‐hr:12‐hr light:dark cycle at constant humidity using standard sugar/yeast/agar (SYA) media (Bass *et al*., 2007). For all the experiments, flies were reared at standard larval density, and enclosing adults were collected over a 12‐h period. Flies were mated for 48 h before sorting into single sexes.

### Drug treatment

β‐GPA (Sigma, St. Louis, MO, USA) was dissolved in ultrapure water and added to SYA food at appropriate concentrations (300, 900, 2700 mm). β‐GPA was added during the food making progress, when the temperature of food is mostly between 40 and 50 °C before food solidification. For control food (0 mm), ultrapure water alone was added. Compound C (Sigma) was dissolved in ultrapure water and added to SYA food at appropriate concentration (200 μm).

### Stress assays

For stress assays, flies were reared and housed as for lifespan experiments. Flies were pretreated with β‐GPA at 900 mm for 30 days and then transferred to food supplemented with 3 m hydrogen peroxide (H_2_O_2_; Sigma) for oxidative stress assays, or to 1.5% agar for starvation assays.

### Dietary restriction

The DR protocol was described in detail in Bass *et al*. (2007).

### Lifespan experiments

Flies were maintained in vials at a density of ten flies per vial. Flies were transferred to new vials every 2 days and scored for deaths. All lifespan experiments have been repeated twice, except starvation lifespan, oxidative stress lifespan, and DR experiments.

### Measurement of LD level

The assay for LD level was performed according to the protocols of the LD kit. The results were expressed as LD equivalents mmol per gprot.

### Measurement of LDH activity

The assay for LDH activity was performed according to the protocols of the LDH kit. The results were expressed as LDH equivalents U per gprot.

### Measurement of food consumption

To measure the amount of food that *Drosophila* consumed, we have performed dye‐calibrated feeding observation as described in Wong *et al*. (2009).

Briefly, 7‐day‐old male and female flies were put on standard SYA food containing 2.5% blue dye (w/w; FD&C Blue No. 1) and 900 mm β‐GPA or ethanol as a control. Feeding was observed for 4 h. The amount of blue dye was determined spectrophotometrically. The relationship between observed feeding events and blue‐dye consumption was analyzed as previously described (Wong *et al*., 2009).

### Western blot analysis

Ten female flies were homogenized in 200 μL of 2× Laemmli loading sample buffer (100 mm Tris 6.8, 20% glycerol, 4% SDS) containing 5% β‐mercaptoethanol. Approximately 40 μg of protein extract was separated by 8% SDS‐PAGE and then transferred to nitrocellulose membranes (transfer buffer: 25 mm Tris, 190 mm glycine, 20% methanol, 0.5% SDS). The membranes were washed in Tris‐buffered saline (TBS; 20 mm Tris‐HCl, pH 7.6, 140 mm NaCl) and blocked with 5% BSA in TBS containing 0.5% Tween‐20 (TBS‐T). Then, membranes were incubated overnight at 4 °C with the following primary antibodies: AMPK (1:500 dilution; CST, San Francisco, CA, USA, #2532), p‐AMPK (1:500 dilution; CST, #2535), ULK1 (Atg1) (1:500 dilution; CST, #8054), p‐ULK1 (p‐Atg1) (1:500 dilution; CST, #5869), Atg5 (1:500 dilution; Novus Biologicals, Denver, CO, USA, NB110‐53818), Atg8 (LC3) (1:200 dilution; CST, #4108), P62 (1:1000 dilution; CST, #5114), S6K (1:500 dilution; CST, #9202), and p‐S6K (1:500 dilution; CST, #9206). Membranes were washed with TBS‐T solution, incubated for 60 min with HRP‐conjugated anti‐rabbit IgG or anti‐mouse IgG (1:5000 dilution; Millipore, Co. Cork, Ireland), washed with TBS‐T, rinsed with double‐deionized water, and immersed in enhanced chemiluminescence‐detecting substrate (SuperSignal West Pico; Pierce Chemical, Rockford, IL USA). Images were captured with Micro Chemi‐DNR Bio‐Imaging Systems (DNR, Mahale HaHamisha, Jerusalen, Israel) or visualized with X‐ray films Kodak, Rochester, NY, USA. The pictures or films were scanned, and the optical density of the bands was determined using nih imagej software (NIH, Baltimore, MD, USA).

### Data analysis

Values were presented as mean ± SEM and analyzed by employing spss (SPSS Inc., Chicago, IL, USA) 10.0 software. The results from lifespan and quantitative Western blot analyses were statistically evaluated using log‐rank test and/or ANOVA followed by Newman–Keuls *post hoc* test. Differences at the *P* < 0.05 level were considered statistically significant. N represents the number of independent biological replicates.

## Author contributions

S.Y. and L.‐H.L designed and performed the experiments, analyzed the data, and wrote the manuscript. J.‐K.Z and D.L. helped in Western blot experiments. F.W. collaborated in analyzing and interpreting the data and developing and editing the manuscript and cowrote the manuscript. J.‐G.C. oversaw the experiments, supervised the project, and wrote and revised the manuscript.

## Funding

No funding information provided.

## Conflict of interests

The authors declare no potential conflict of interests.

## Supporting information


**Fig. S1** Feeding behaviour ofβ‐GPAtreatment *Drosophila*.
**Fig. S2** AMPK activityin 300 mm β‐GPAtreatment*Drosophila*.
**Fig. S3** AMPK activityin *Drosophila*after 900 mm β‐GPAtreated for 50 days.
**Fig. S4** β‐GPAincreases lifespan irrespective of foodconcentration
**Fig. S5** β‐GPATreatment of *Drosophila* downregulates S6Kactivity.
