# Supplementary material for: β‐Guanidinopropionic acid extends the lifespan of Drosophila melanogaster via an AMP‐activated protein kinase‐dependent increase in autophagy
Source: Aging Cell. 2015 Jun 29;14(6):1024–33. doi: 10.1111/acel.12371 (PMC4693457; doi:10.1111/acel.12371)
Supplement: Supplementary file 1 — Fig. S1 Feeding behaviour ofβ‐GPAtreatment Drosophila. Fig. S2 AMPK activityin 300 mm β‐GPAtreatmentDrosophila. Fig. S3 AMPK activityin Drosophilaafter 900 mm β‐GPAtreated for 50 days. Fig. S4 β‐GPAincreases lifespan irrespective of foodconcentration Fig. S5 β‐GPATreatment of Drosophila downregulates S6Kactivity. [file ACEL-14-1024-s001.pdf]

Supplemental Information

Supplementary Figures

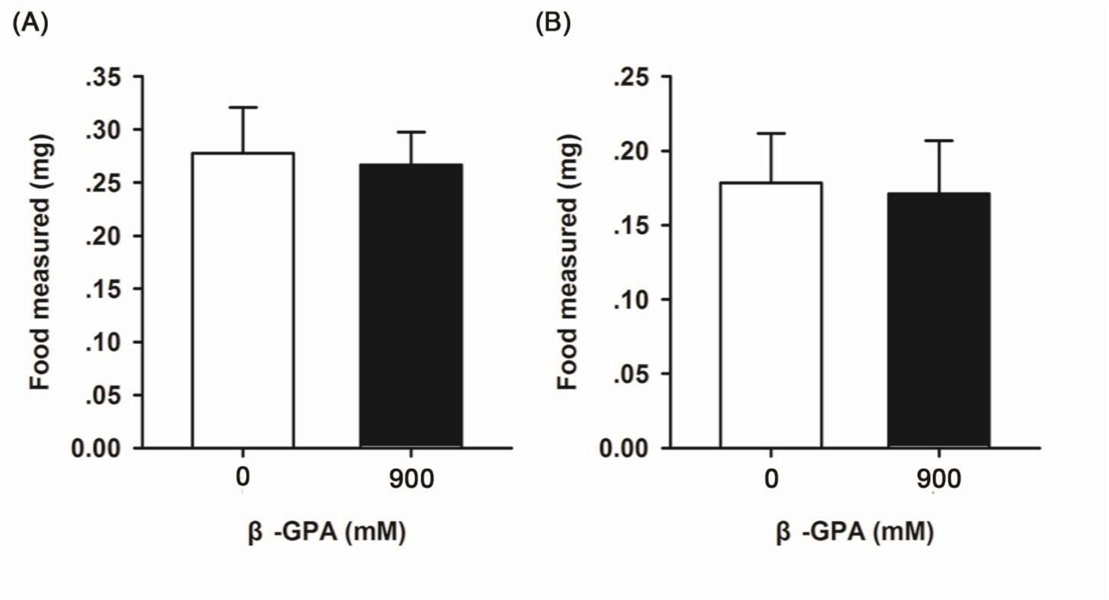

Figure S1. Feeding behaviour of β-GPA treatment Drosophila

(A-B) Figure represents feeding observations during a 4 hours period. There was no significant difference in the feeding behavior of flies on β-GPA food compared to flies on food not containing β-GPA (n=6, t test, *$p < 0.05$ vs control). Data are shown as mean ± SEM.

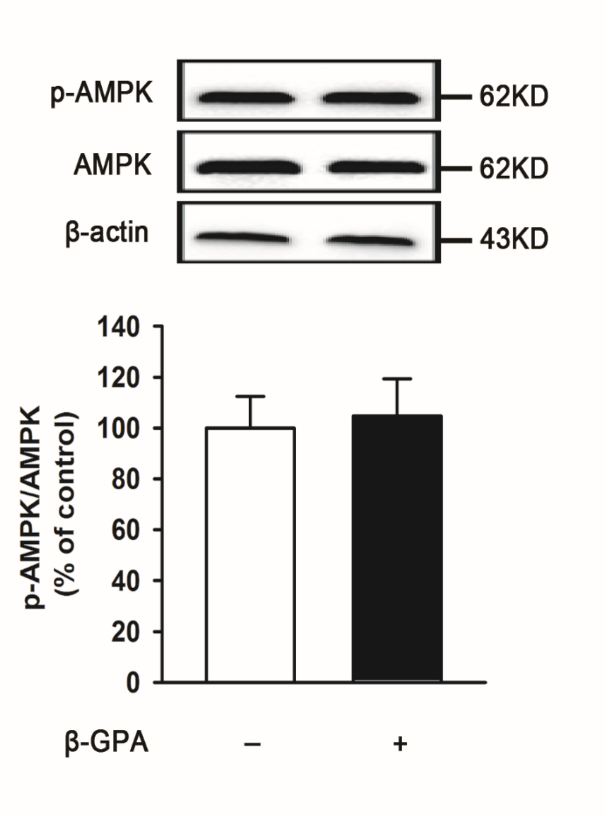

Figure S2. AMPK activity in 300 mM β-GPA treatment Drosophila

Western blot analysis of p-AMPK, AMPK in WT flies. There is no effect of β-GPA on phosphorylation of AMPK at doses of 300 mM (n=6, t test, *$p < 0.05$ vs control). Data are shown as mean ± SEM.

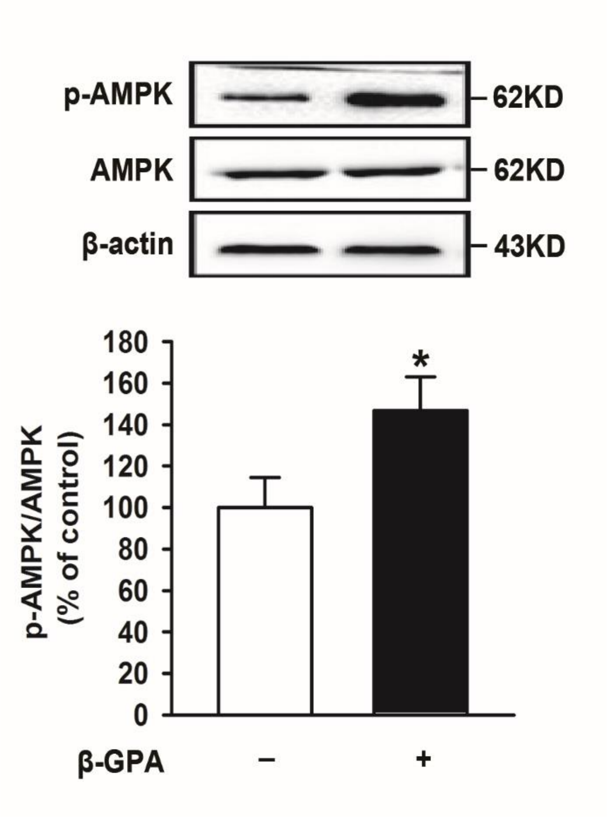

Figure S3. AMPK activity in Drosophila after 900 mM β-GPA treated for 50 days.

Western blot analysis of p-AMPK, AMPK in WT flies. We found 900mM β-GPA can increase AMPK activity after 50 days. (n=6, t test, *p < 0.05 vs control). Data are shown as mean ± SEM.

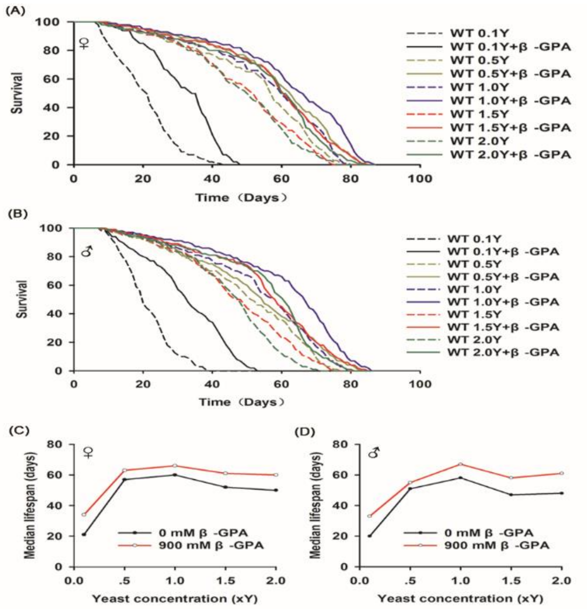

Figure S4. β-GPA increases lifespan irrespective of food concentration

(A-B) Complete survival curves for the dietary restriction (DR) experiment that is presented in Figure 6. Plotted are survival curves for WT females and males against yeast concentration (0.1x, 0.5x, 1.0x, 1.5x, and 2.0x yeast) in SYA food (dashed line) and on the same food concentrations but supplemented with 900 mM β-GPA (solid line). Flies on 900 mM β-GPA food had significantly increased lifespan at each yeast concentration. (C-D) Median lifespan across different yeast concentrations in SYA food. (n=200, $p < 0.0001$ vs control, log-rank test)

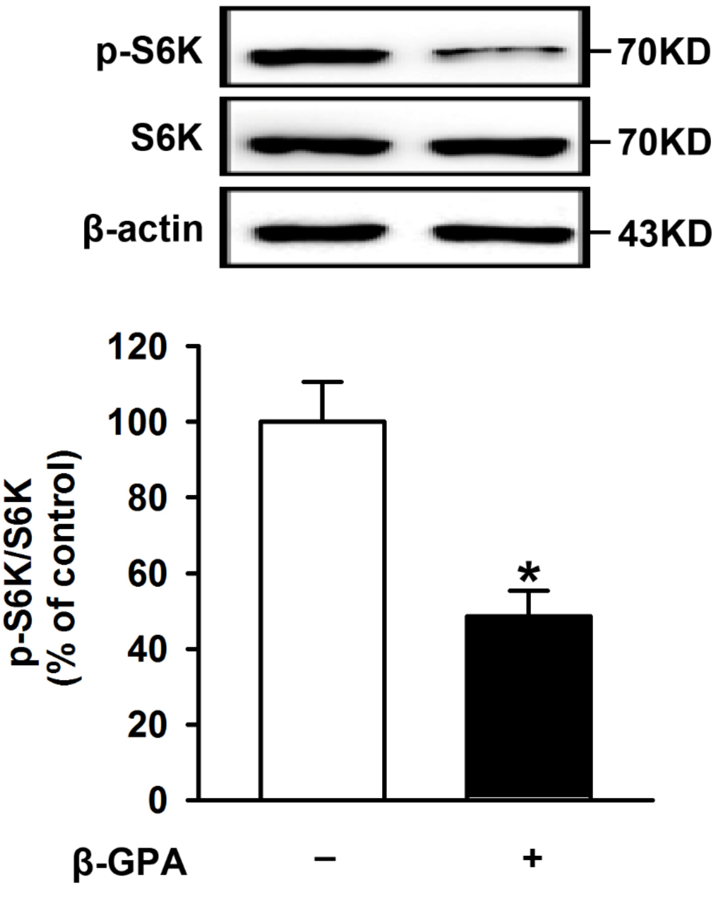

Figure S5. β-GPA Treatment of Drosophila downregulates S6K activity.

Western blot analysis of p-S6K, S6K in WT flies. β-GPA was found to efficiently decreases levels of p-S6K in flies (n=6, t test, *$p < 0.05$ vs control). Data are shown as mean ± SEM.
